# Untargeted Metabolomics Identifies Biomarkers for MCADD Neonates in Dried Blood Spots

**DOI:** 10.3390/ijms24119657

**Published:** 2023-06-02

**Authors:** Rajaa Sebaa, Maha AlMogren, Wafaa Alseraty, Anas M. Abdel Rahman

**Affiliations:** 1 Department of Medical Laboratories, College of Applied Medical Sciences, University of Shaqra, Al-Dawadmi 17472, Saudi Arabia; r.sebaa@su.edu.sa; 2 Metabolomics Section, Department of Clinical Genomics, Center for Genomics Medicine, King Faisal Specialist Hospital and Research Centre (KFSHRC), Riyadh 11211, Saudi Arabia; malmogren@alfaisal.edu; 3 Department of Biochemistry and Molecular Medicine, College of Medicine, Al Faisal University, Riyadh 11533, Saudi Arabia; 4 Department of Nursing, College of Applied Medical Sciences, University of Shaqra, Al-Dawadmi 17472, Saudi Arabia; walseraty@su.edu.sa

**Keywords:** MCADD, DBS, newborns, untargeted metabolomics, mass-spectrometry, metabolic biomarkers, oxidized lipids, glutathione

## Abstract

Medium-chain acyl-CoA dehydrogenase deficiency (MCADD) is the most common inherited mitochondrial metabolic disease of fatty acid β-oxidation, especially in newborns. MCADD is clinically diagnosed using Newborn Bloodspot Screening (NBS) and genetic testing. Still, these methods have limitations, such as false negatives or positives in NBS and the variants of uncertain significance in genetic testing. Thus, complementary diagnostic approaches for MCADD are needed. Recently, untargeted metabolomics has been proposed as a diagnostic approach for inherited metabolic diseases (IMDs) due to its ability to detect a wide range of metabolic alterations. We performed an untargeted metabolic profiling of dried blood spots (DBS) from MCADD newborns (*n* = 14) and healthy controls (*n* = 14) to discover potential metabolic biomarkers/pathways associated with MCADD. Extracted metabolites from DBS samples were analyzed using UPLC-QToF-MS for untargeted metabolomics analyses. Multivariate and univariate analyses were used to analyze the metabolomics data, and pathway and biomarker analyses were also performed on the significantly identified endogenous metabolites. The MCADD newborns had 1034 significantly dysregulated metabolites compared to healthy newborns (moderated *t*-test, no correction, *p*-value ≤ 0.05, FC 1.5). A total of 23 endogenous metabolites were up-regulated, while 84 endogenous metabolites were down-regulated. Pathway analyses showed phenylalanine, tyrosine, and tryptophan biosynthesis as the most affected pathways. Potential metabolic biomarkers for MCADD were PGP (a21:0/PG/F1alpha) and glutathione, with an area under the curve (AUC) of 0.949 and 0.898, respectively. PGP (a21:0/PG/F1alpha) was the first oxidized lipid in the top 15 biomarker list affected by MCADD. Additionally, glutathione was chosen to indicate oxidative stress events that could happen during fatty acid oxidation defects. Our findings suggest that MCADD newborns may have oxidative stress events as signs of the disease. However, further validations of these biomarkers are needed in future studies to ensure their accuracy and reliability as complementary markers with established MCADD markers for clinical diagnosis.

## 1. Introduction

Medium-chain acyl-CoA dehydrogenase deficiency (MCADD) is one of the inherited metabolic disorders (IMDs) that are associated with metabolic disturbances [1]. MCADD is the most common inherited fatty acid *β*-oxidation disorder, and it is caused by various genetic mutations in the acyl-CoA dehydrogenase medium chain (*ACADM*) gene. It encodes a mitochondrial enzyme called medium-chain acyl-CoA dehydrogenase, located on chromosome 1p31 in an autosomal recessive manner. The manifestation of MCADD is contingent upon the inheritance of mutated alleles from both parents. Homozygous mutations can be acquired through consanguineous unions or a random mutation in the second allele in heterozygous parents [2]. In populations with higher consanguinity rates, the incidence of autosomal recessive diseases increases 50-fold [3]. For instance, in eastern Saudi Arabia, consanguinity rates are as high as 40% among first cousins, and up to 60% in intermarriages between relatives [4,5]. 

The birth prevalence of MCADD has been estimated in North America and Northern Europe to be approximately 1:5000 to 1:20,000 [6]. Another study reported that the estimated incidence of MCADD in different populations, such as Caucasians, is 1:8000–1:20,000; in Germany, it is 1:4900–1:8500; and in the United States, it is 1:10,000–30,000 [7,8]. In contrast, the incidence of MCADD is relatively low among Asians, with estimations of 1:100,000 in Japan, and China was reported at 1:80,332–1:282,591 [9], indicating that ethnic and regional differences significantly and differently impact the incidence of MCADD. In Saudi Arabia, MCADD has a prevalence of 1/18,000 [4,10]. Importantly, the prevalence of MCADD only accounts for patients with obvious symptoms who had clinical visits and underwent clinical examinations without including the suspected MCADD patients without signs and at high risk. Thus, the true incidence of MCADD should not be underestimated and requires accurate investigation. 

Phenotypically, MCADD patients, particularly newborns, vary in their clinical manifestations, which range from mild-to-severe symptoms, such as hypoglycemia and cardiomyopathy. However, a growing number of asymptomatic or pre-symptomatic newborns with MCADD make the diagnosis of MCADD challenging [11,12]. 

At the cellular level, fatty acids with various lengths enter mitochondria for the *β*-oxidation pathway. Medium-chain fatty acids can enter mitochondria through membrane diffusion in contrast with long-chain fatty acids requiring the carnitine shuttle system for transporting. Once medium-chain fatty acids are transported into the mitochondria matrix, they are activated into their corresponding medium of fatty acyl-CoAs, which are targeted and oxidized by medium-chain acyl-CoA dehydrogenase—an enzyme involved in the mitochondrial fatty acid *β*-oxidation to produce acetyl-CoA—thus, reducing agents and ATP in the mitochondrial matrix [13]. Defects or absences of the enzyme medium-chain acyl-CoA dehydrogenase, as in MCADD, lead to the accumulation of medium fatty acyl-CoAs in the mitochondria matrix, which subsequently binds to the mitochondrial carnitine molecules, resulting in the formation of medium acylcarnitines. The latter need to be eliminated from the mitochondrial matrix and released into circulation as medium acylcarnitines [12].

In the MCADD condition, there is an abnormal accumulation of acylcarnitines with a length of (C6-C12) in circulation [14]. For that reason, the concentration of octanoylcarnitine (C8) and decanoylcarnitine (C10), and their ratios C8/C10 and C8/C2, are mostly used as MCADD markers for the purpose of diagnosis [15]. 

Early diagnosis of MCADD helps alleviate the disease complications and improves affected newborns’ health outcomes by providing proper treatments and interventions. MCADD diagnosis is achieved using Newborn Bloodspot Screening (NBS) and genetic testing. NBS measures the MCADD markers (i.e., C8, C8/C10, C8/C2, and acylcarnitines) through tandem mass spectrometry [16]. However, the NBS programs’ efficiency in detecting IMDs, including MCADD, shows drawbacks concerning clinical diagnosis. To illustrate, NBS could miss certain babies with IMDs, called false-negative results, which delay the detection of IMDs and can cause health implications [17,18,19]. In addition, NBS has been associated with false-positive results for IMDs in babies who are not truly affected, causing overuse of healthcare services, as well as stress and anxiety for the parents of the suspected babies [20,21,22]. A Canadian study has reported several MCADD infants diagnosed as false positive; such a fact significantly impacts the health system by increasing the demand for unneeded health services for those who are not truly MCADD-affected infants [23]. Thus, a study suggested utilizing the genetic sequencing of DBS from false-positive-diagnosed MCADD patients as a validation method for the diagnosed patients [24]. 

Therefore, the positive NBS results for MCADD are confirmed using molecular genetic testing, such as whole or exosome sequencing, which detects mutations in the *ACADM* gene. Abnormal newborn screening results are linked to 54 variants of uncertain significance (VUS), previously unknown, in the *ACADM* gene [25]. Undoubtedly, VUS is a dilemma for clinicians because they could be misleading in diagnosing MCADD due to the lack of information about these unknown mutations and whether they are functionally related to MCADD. Based on these drawbacks associated with the NBS program and genetic approaches, there have been demands in the clinical field to identify other alternative and complementary diagnostic approaches for MCADD. 

Untargeted metabolomics, which measures small metabolites, has recently emerged as a promising diagnostic tool for IMDs due to its exceptional ability to detect a broad range of the altered metabolites affected by these disorders. This approach allows for identifying IMD-specific biomarkers and pathways [26,27]. Only one metabolomics study has explored the metabolic changes in dried blood spots (DBS) that were collected from MCADD, and it revealed abnormal levels of oxidized phosphatidylcholines in MCADD patients [28]. In order to have a better understanding of the underlying mechanisms of MCADD, and to discover new potential metabolic biomarkers for MCADD, more metabolomics studies are required; these studies will help in the clinical diagnosis of MCADD and improve the drawbacks found in the current diagnostic approaches. Thus, we aimed to investigate potential metabolic biomarkers and pathways for MCADD by using DBS samples from MCADD newborns and healthy newborns to be analyzed by a high-throughput untargeted metabolomics approach.

## 2. Results

### 2.1. Demographic Data

The demographic data of MCADD and healthy control newborns are summarized in Table 1. The LC-MS-based acylcarnitine data of MCADD and healthy newborns generated from the NBS program were used and carefully analyzed for DBS sample selection. The LC-MS-based measurements of the acylcarnitine panel were specifically for C8-carnitine, C6-carnitine, the C8\C10-carnitine ratio, and the C8\C2-carnitine ratio. Then, the DBS cards from MCADD (*n* = 14), as well as from the age- and gender-matched healthy controls (*n* = 14) were collected from the metabolomics section lab for metabolomics profiling. Additionally, we considered the age of the participants to be at an early age. Thus, the average age for the MCADD group and healthy control were 15.3 ± 11.0 and 11 ± 9.4, respectively. MCADD newborns were not diagnosed with other diseases and received no treatment at the sample collection stage.

### 2.2. Metabolomics Profiling of MCADD Newborns

**Figure 1:**
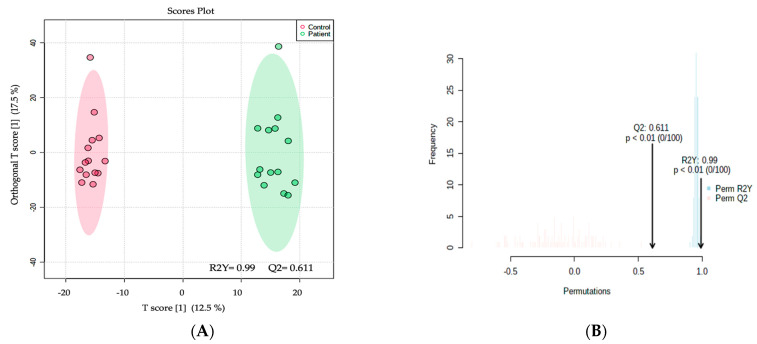
(**A**) The orthogonal partial least squares-discriminant analysis (OPLS-DA) displays a clear separation between the two groups (MCADD newborns vs. healthy controls). The robustness of the created models was evaluated by the fitness of the model (R2Y = 0.99) and predictive ability (Q2 = 0.611) values in a larger dataset (*n* = 100). (**B**) The permutation analysis showing the observed and cross-validated R2Y and Q2 coefficients.

A total of 17,542 mass ion features were detected—11,318 in positive and 6224 in negative ionization modes (Table S1). Features with missing values of >80% were excluded (22.2%), thus 13,500 features remained for further statistical analysis. The results from the orthogonal partial least squares-discriminant analysis (OPLS-DA) are displayed in Figure 1A; these results show a significant difference between the two groups (MCADD newborns and healthy controls) with an R2Y = 0.99 and Q2 = 0.611, indicating a significant metabolic difference between the two groups. The permutation analysis (Figure 1B) shows the observed and cross-validated R2Y and Q2 coefficients.

**Figure 2:**
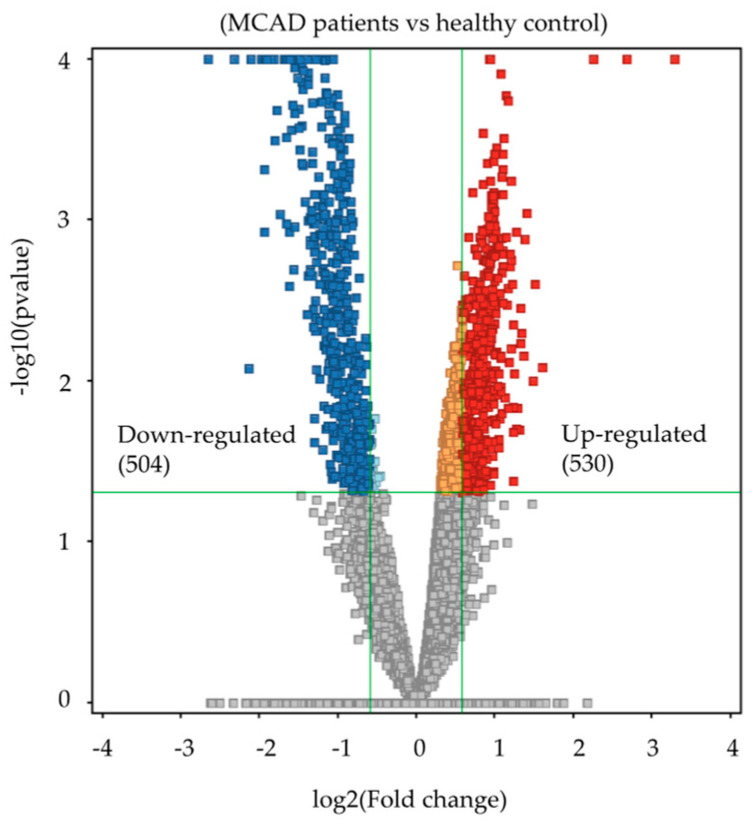
The volcano plot analysis showing significantly dysregulated metabolites between the two groups. (Moderated *t*-test, raw *p*-value ≤ 0.05, fold change (FC) 1.5). A total of 530 metabolites were up-regulated (red) and 504 were down-regulated (blue) in MCADD newborns.

**Figure 3:**
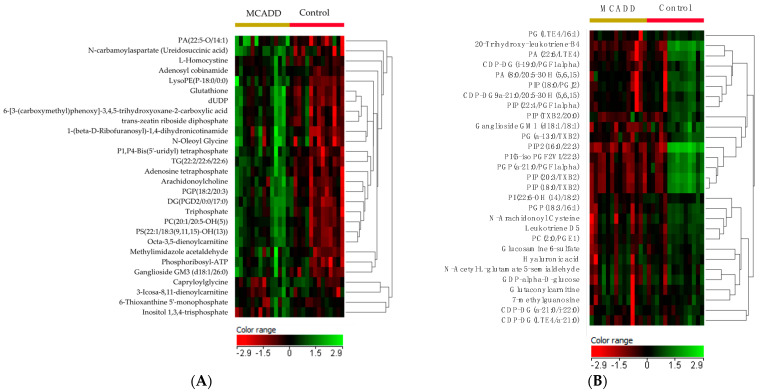
(**A**) Heatmap demonstrating the up-regulated metabolites and (**B**) down-regulated metabolites in MCADD newborns compared with healthy controls. Red indicates down-regulated metabolites, and green indicates up-regulated metabolites.

A univariate analysis was conducted to identify the significantly different features between the two groups. A volcano plot analysis was conducted, and the 13,500 features between the two groups were evaluated. (Moderated *t*-test, raw *p*-value ≤ 0.05, FC 1.5). These features showed 1034 significantly dysregulated metabolites (Table S2), 504 up-regulated, and 530 down-regulated in newborns with MCADD (Figure 2). A heatmap of the 107 identified endogenous metabolites is displayed in Figure S1. Heatmaps of the significantly dysregulated up- and down-regulated metabolites in MCADD newborns and healthy controls are shown in Figure 3A,B.

A total of 360 significantly dysregulated metabolites were identified (Table S3). In total, 107 endogenous metabolites remained after excluding exogenous compounds (drugs, drug metabolites, environmental exposures, etc.), and these were retained for the analyses of pathways and biomarkers (Table S4).

### 2.3. Metabolomics Pathway Analysis

**Figure 4:**
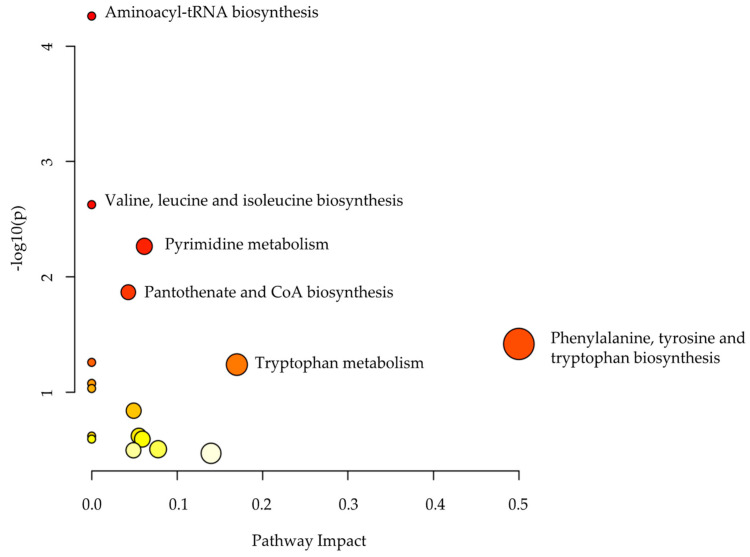
Pathway analysis of the significantly endogenous dysregulated metabolites in MCADD newborns and healthy controls.

Pathway analysis was performed on the significantly dysregulated metabolites (*n* = 107) to identify the most altered pathways. The most affected pathways between the two groups were phenylalanine, tyrosine, and tryptophan biosynthesis (Figure 4). 

### 2.4. Biomarker Analysis

**Figure 5:**
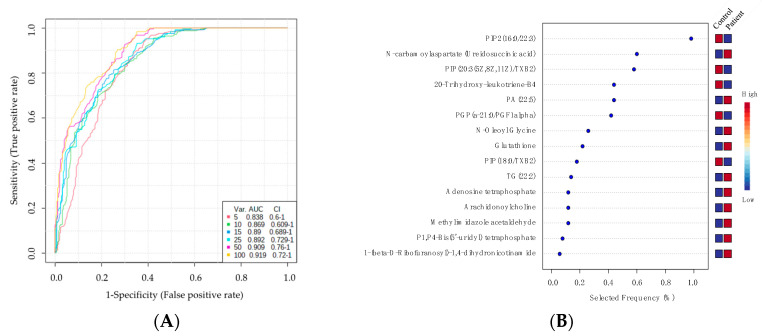

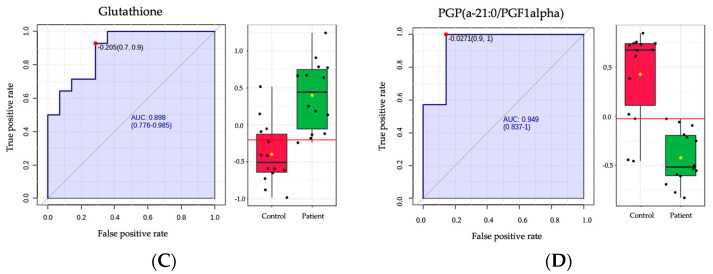
Metabolomics profiling and biomarker evaluation between MCADD newborns and healthy controls. (**A**) Receiver operating characteristics (ROC) curve for significantly dysregulated metabolites in MCADD newborns. (**B**) Frequency plot of 15 identified metabolites. (**C**,**D**) Representative ROC curves for two significantly dysregulated metabolite molecules (glutathione, AUC = 0.898, and PGP (a21:0/PGF1alpha), AUC = 0.949) in MCADD newborns.

A receiver operating characteristic (ROC) curve analysis (Figure 5A) was created; this was achieved by using PLS-DA as a classification and feature ranking approach to evaluate potential biomarkers. Figure 5B shows a frequency plot of 15 identified metabolites. Figure 5C,D show the glutathione (AUC = 0.898) and PGP (a-21:0/PGF1alpha) (AUC = 0.949) that were up- and down-regulated in MCADD newborns compared to healthy controls, respectively. The top 15 dysregulated metabolites with their AUC are mentioned in (Table S5).

## 3. Discussion

### 3.1. Untargeted Metabolomics as a Complementary Diagnostic Approach for MCADD 

Due to the limitations of the current diagnostic approaches—including NBS and genetic testing—and the demands in the clinical field to identify a complementary approach for the diagnosis of MCADD, few studies have advised—by highlighting the potential altered metabolic pathways and biomarkers associated with the disease—on the great ability of untargeted metabolomics to diagnose MCADD [26,28,29]. Similarly, our study has fully exploited the benefits and capabilities of untargeted metabolomics to identify metabolic biomarkers/pathways for MCADD. Our metabolomics study demonstrated interesting findings. MCADD newborns had 1034 dysregulated metabolites, of which 107 endogenous identified metabolites were altered, and 23 and 84 were up-regulated and down-regulated, respectively. Moreover, the most affected metabolic pathways in MCADD newborns were the phenylalanine, tyrosine, and tryptophan pathways. Cellular oxidative stress and defense-mechanism-related metabolites such as oxidized lipids and glutathione were significantly affected. Some of our findings have been reported previously [1,28]; however, for the first time, we reported that the DBS from MCADD newborns had elevated glutathione and had altered certain types of oxidized lipids, notably those not reported before, which is suggestive of increased oxidative stress events. These findings may be used as potential biomarkers for MCADD as a complementary diagnostic approach in addition to the most currently known acylcarnitine biomarkers. 

### 3.2. Dysregulated Amino Acids Resulted from the Defective Mitochondrial Oxidation of Medium-Chain Fatty Acid in MCADD Newborns

The study findings showed that several amino acids were dysregulated in MCADD newborns. Furthermore, multiple amino acid-related pathways, including phenylalanine, tyrosine, and tryptophan biosynthesis, were altered in MCADD. Generally, it is well accepted that glucose, fatty acids, and amino acids are the substrates required to preserve the metabolic homeostasis in living organisms. Amino acid homeostasis fundamentally differs from carbohydrate and lipid homeostasis in the human body and, except in endogenous proteins, amino acids have no independent storage form [30]. Endogenous amino acids that are biosynthetically made are the primary substrates for hepatic gluconeogenesis, which requires a consistent supply of acetyl-CoA being produced from fatty acid oxidation [31], indicating the important link between amino acids and fatty acids. Due to this link, the altered amino acid levels in MCADD newborns could be explained by the defects in fatty acid oxidation and the reduction in acetyl-CoA production, which affect the homeostasis of amino acids.

As we found that several amino acids are affected by the condition of MCADD, proline—a non-essential amino acid—was down-regulated in the metabolic profile of the MCADD newborns. This compound plays a part in protein structure and function, and it maintains cellular redox homeostasis [30]. Previously, studies have shown the role of proline in maintaining redox homeostasis [32]. Furthermore, several pathological conditions have been connected to the dysregulation of tryptophan biosynthesis. It has also been shown that metabolites related to the tryptophan pathway may influence the function of mitochondria and the redox status [33], which is consistent with our metabolomics data; this reveals that the tryptophan in MCADD newborns was altered. Taken together, amino acids that were affected in MCADD newborns are probably linked to the altered fatty acid oxidation process and the defective mitochondrial redox status in the context of MCADD. 

### 3.3. Distinctive Lipid Patterns Observed in MCADD Newborns

The accumulation of acylcarnitines accompanies MCADD. Thus, medium acylcarnitines, including octanoylcarnitine (C8) and decanoylcarnitine (C10), and the ratios of C8/C2 and C8/C10, are used as well-known biomarkers for MCADD [15]. However, these biomarkers have been reported as false-positive results in some MCADD cases [23], which has pushed researchers toward finding other acylcarnitine species to diagnose MCADD. Few studies have focused on identifying the new acylcarnitine species or lipid metabolites used for MCADD-affected patients. A study that used plasma samples from MCADD patients for untargeted high-throughput metabolomics analyses revealed certain distinctive acylcarnitines related to MCADD, including L-Hexanoylcarnitine and 2-trans,4-cis-Decadienoylcarnitine [26]. Consistent with previous findings, our metabolomics analyses of DBS discovered other acylcarnitine species, including octa-3,5-dienoylcarnitine, glutaconylcarnitine, and (8Z,11Z)-3-Icosa-8,11-dienoylcarnitine. Not only were the acylcarnitines declared to be altered in the context of MCADD, but other lipids species can also be affected (as explained below), such as oxidized phospholipids, CDP-diacylglycerols, and cardiolipin.

Dysfunctional mitochondria, as seen in the MCADD condition, can be correlated with the excessive production of the reactive oxygen species (ROS) oxidants, which can be subsequently viewed as a leading cause of oxidative stress. The latter occurs when there is an imbalance between the ROS production and the antioxidant defense system in the cells that represent increased ROS production, as well as due to the decreased antioxidants involved in defense mechanisms. The increased level of ROS can target several biological molecules, including lipids, with their various classes. Membrane phospholipids are ROS targets. Our study is the second that has used untargeted metabolomics for the DBS samples from MCADD newborns. One of the hallmarks observed in our data is that different oxidized lipids, including oxidized phosphocholines, oxidized phosphatidylserines, and oxidized CDP-diacylglycerols, were detected and altered; these results are suggestive of the increased oxidative stress that occurs in the MCADD condition. Our data are consistent with the previously published data that used targeted metabolomics analyses on DBS from MCADD patients, and which also showed elevated oxidized phospholipids, particularly oxidized phosphatidylcholines [28]. Our study has further highlighted oxidized lipids, including oxidized phosphatidylserines and CDP-diacylglycerols, as the altered lipid species that are found in MCADD.

Additionally, cardiolipin is a type of phospholipid embedded in the inner mitochondrial membrane (IMM), where an oxidative phosphorylation system takes place to produce cellular energy in the form of ATP [34,35]. Additionally, cardiolipin is important for mitochondrial morphology and dynamics, which are required to support mitochondrial function and bioenergetics. Indeed, cardiolipin alterations are associated with dysfunctional mitochondria, as is seen in the pathologies of certain diseases [36,37]. In addition, as cardiolipin is naturally composed of four fatty acids and glycerol forming its tight composition for ultimate function, the cellular and mitochondrial lipid changes that occur in rare metabolic and mitochondrial diseases could affect the composition of cardiolipin, which can, in turn, decrease cardiolipin function and production. Experimentally, it was evident that the mitochondrial function was defective in MCADD0-patient-derived skin fibroblasts as they represented declined oxidative phosphorylation-system-related proteins and oxygen consumption [38]. To our knowledge, in line with the previous facts, we are the first to report that cardiolipin is decreased in the metabolomics profiling of DBS from MCADD newborns, revealing the pathological impact of MCADD on the reduced abundance of cardiolipin, which may contribute to the observed mitochondrial dysfunction in MCADD in previous studies [38].

Moreover, based on the biomarker analyses, certain oxidized lipids were altered, including PGP (a-21:0/PGF1alpha), which was an oxidized lipid molecule in the top 15 biomarkers, and could be used as potential biomarkers for MCADD. However, further validation experiments could be conducted using targeted metabolomics analyses of DBS samples from MCADD to verify its usage as an MCADD biomarker. 

### 3.4. Elevated Glutathione, a Non-Enzymatic Antioxidant Defense System, Found in MCADD Newborns

Glutathione is a tripeptide composed of glutamate, cysteine, and glycine residues that are joined by γ-peptidic bonds through the action of the glutamate–cysteine ligase and GSH synthetase. Glutathione is a non-enzymatically antioxidant defense system involved in several cellular compartments, including mitochondria. It plays an important role in detoxifying the ROS produced in cells to protect against lipid, protein, and DNA damage, which can lead to the development of diseases [39]. Interestingly, our untargeted metabolomics profiling of DBS from MCADD newborns showed that the glutathione level is significantly elevated compared with the healthy controls. This finding could be explained by the notion that glutathione might be used as a compensatory mechanism in MCADD newborns to protect against the oxidized lipids; this find was also found in our metabolomics data, and could potentially alleviate the symptoms of oxidative stress. In 2004, a study was conducted by Koruk et al. to investigate the status of oxidative stress and antioxidant enzymes in the development of nonalcoholic steatohepatitis (NASH), which is in part associated with mitochondrial dysfunction and lipid accumulations [40]. Their results showed that the serum level of glutathione in NASH patients was significantly increased compared with the controls, indicating the involvement of glutathione in this pathological condition [41].

In contrast, an animal study, which conducted in vitro experiments, focused on understanding the pathological mechanisms involved in the neurologic symptoms in MCADD patients. Their study used rat cerebral cortex homogenates treated with octanoate (OA) and decanoate (DA) to mimic the real brain pictures of MCADD-affected patients with accumulated OA and DA. Their results showed that the treated brain homogenates had decreased glutathione levels [42]. However, the contradictory findings of the in vitro study could be explained based on the following fact. The in vitro experiment does not mimic the complex interplay between oxidative stress and glutathione in the living cellular and whole system of MCADD patients, which is more complicated than those found in vitro studies. For example, in vitro experiments must account for the many biological factors and regulators in living organisms that can impact the glutathione level. In comparison, between our data and previously published data, for the first time, we found that glutathione is elevated in MCADD, which is attributed to the expected oxidative stress events during the diseases. Thus, after further validation studies, glutathione could also be used as a potential metabolic biomarker for MCADD. 

As mentioned previously, the current diagnostic tests for MCADD, including genetic sequencing and NBS, have some limitations causing inaccuracy in the diagnosis of MCADD due to the existence of VUS and false positive/negative results. As an alternative approach, untargeted metabolomics offers great opportunities for MCADD diagnosis because it can detect thousands of altered metabolites within a very tiny number of biological samples, thus reflecting the pathological status of the disease, that are collected from MCADD patients. Focusing on these altered metabolites gives a high potential to identify new metabolic biomarkers that could be correlated with MCADD disease in addition to the current limited acylcarnitine markers. For these reasons, our study used an untargeted metabolomics approach to analyze the samples collected from MCADD patients to discover new potential biomarkers that could be used for diagnosis in the future. However, to ensure the specificity of the biomarkers, they needed to be validated by larger, independent cohorts of MCADD and other types of metabolic diseases from separate studies. Additionally, targeted metabolomics approaches can measure our newly discovered metabolic biomarkers in MCADD samples. In addition, other omics studies are required to comprehensively understand MCADD patients’ pathophysiology. Omics-based measurements can provide insights regarding the changes in several of the biological molecules involved in genomics, transcriptomics, proteomics, metabolomics, epigenomics, epitranscriptomics, epiproteomics, and—recently—redox omics [43,44]. Moreover, the biological DBS samples were used for our metabolomics studies and needed to be compared with other sample types, including plasma and urine samples, which ultimately expanded information about the suitable sample type for the proper diagnostic tests and helped to design the exact diagnosis process for MCADD. Following the suggestions mentioned above, more accurate predictive diagnostic approaches could be utilized to diagnose MCADD, which help to determine the proper treatments given to the affected newborns for longer survival and greater health outcomes.

## 4. Materials and Methods

### 4.1. Ethical Approval

The Institutional Review Boards at King Faisal Specialist Hospital and Research Centre (KFSHRC) in Riyadh, Saudi Arabia (RAC # 2160027) reviewed and approved this study and its related procedures. 

### 4.2. Biological Samples

DBS samples were obtained from the metabolomics section in the Center for Genomic Medicine at King Faisal Specialist Hospital and Research Center (KFSHRC). The samples were collected from MCADD newborns (*n* = 14) and healthy newborns (controls) (*n* = 14). These newborns were age- and gender-matched. The inclusion criteria for the patient group included newborns that were positively diagnosed, through the newborn screening program’s platform, with only MCADD. For the control group, the inclusion criteria were healthy, gender-, and age-matched newborns. Additionally, newborns who were less than a month old were included as the average age of the MCADD newborns was 15.3 days, and for healthy newborns it was 11 days. Any DBS samples collected from newborns diagnosed with other IMDs or were older than a month were excluded. 

### 4.3. Chemicals and Materials

LC-MS grade water, acetonitrile (ACN), methanol, and formic acid, were purchased from Fisher Scientific (Ottawa, ON, Canada). 

### 4.4. Sample Preparation

The metabolites were extracted as reported before with some modifications [45]. In detail, one punch, at a 3.2 mm size, was collected from each DBS sample and transferred into a 96-well plate for metabolite extraction. Metabolite extraction was performed by adding 250 µL of extraction solvent (20:40:40) (H_2_O: ACN: MeOH) to each well with agitation for 2 h at room temperature. Subsequently, the sample extracts were dried using SpeedVac Thermo Fischer, (Christ, Germany). The dried samples were reconstituted in 100 µL of 50% A: B mobile phase (A: 0.1% Formic acid in H_2_O, B: 0.1% FA in 50% ACN: MeOH). Additional punches were taken for quality control (QC) from the project samples to maintain the instrument performance. All study and quality control samples were placed on the UPLC-QToF-MS autosampler for metabolomics analyses. The quality control samples were analyzed once after 10 study samples for the metabolomics analyses.

### 4.5. LC-MS Metabolomics 

The metabolomics analyses were conducted with a Waters Acquity UPLC system coupled with a Xevo G2-S QTOF mass spectrometer (which was equipped with an electrospray ionization source (ESI) [45,46]). In detail, the extracted metabolites were chromatographed using an ACQUITY UPLC using a XSelect (100 × 2.1 mm, 2.5 μm) column (Waters Ltd., Elstree, UK). The mobile phase was composed of 0.1% formic acid in distilled water (dH2O), as solvent A and solvent B consisted of 0.1% formic acid in 50% ACN: MeOH. A gradient elution schedule was run at a 300 μL/min flow rate as follows: 0–16 min 5–95% A, 16–19 min 5% A, 19–20 min 5–95% A, and 20–22 min 95–95% A. The MS spectra were acquired separately under positive and negative electrospray ionization modes (ESI+, ESI−). The MS conditions were as follows: source temperature was 150 °C, the desolvation temperature was 500 °C (ESI+) or 140 (ESI−), the capillary voltage was 3.20 kV (ESI+) or 3 kV (ESI−), cone voltage was 40 V, desolvation gas flow was 800.0 L/h, and cone gas flow was 50 L/h. The collision energies of the low and high functions were set, in MSE mode, at 0 and 10–50 V, respectively. The mass spectrometer was calibrated with sodium formate in 100–1200 Da. Data were collected in continuum mode with a Masslynx™ V4.1 (Waters Technologies, Milford, MA, USA) workstation. 

### 4.6. Data Processing and Statistical Analyses 

The MS raw data were processed following a standard pipeline, starting from an alignment based on the m/z value and the ion signals’ retention time, and the peak picking and signal filtering were based on peak quality using the Progenesis QI v.3.0 software from Waters (Waters Technologies, Milford, MA., USA). Features detected in at least 80% of the samples were retained for further analyses. Multivariate statistical analysis was performed using MetaboAnalyst version 5.0 (McGill University, Montreal, Canada) (http://www.metaboanalyst.ca, accessed on 5 January 2023) [47]. For proper selection of the right statistical model, the datasets (compounds and abundances) were mean-normalized, Pareto-scaled, and log-transformed to maintain their normal distribution. The normalized datasets generated partial least squares-discriminant analysis (PLS-DA) and orthogonal partial least squares-discriminant analysis (OPLS-DA) models. OPLS-DA models created were evaluated using the fitness of model (R2Y) and predictive ability (Q2) values with a permutation validation of 100 samples. Univariate analysis was performed using Mass Profiler Professional software (Agilent Inc., Santa Clara, CA, USA) [48]. In addition, volcano plots were used to identify, using MPP Software, the significantly altered mass features, which were based on a fold change (FC) cut-off of 1.5 and a no correction *p* value < 0.05. The heatmap analysis for the altered features was performed using the distance measure of Pearson. Pathway analysis, biomarkers linked with MCAD disorder, and receiver operating characteristic (ROC) curves were created using the PLS-DA approach in MetaboAnalyst v 5.0 for the purpose of global analysis, which was conducted to identify possible biomarkers.

### 4.7. Peak Annotation (Metabolite Identification) 

The significant features in each dataset were selected and tagged in ProgensisIQ software for peak annotation. The chemical structures of the metabolites were identified by acquiring their accurate precursor masses, fragmentation pattern, and isotopic distribution to the Human Metabolome Database (HMDB) [49]. The precursor mass and theoretical MS/MS fragmentation tolerance values were set to 12 ppm. The exogenous compounds, such as drugs, food additives, and environmental compounds, were excluded manually from the final list.

## 5. Conclusions

Our metabolomics findings have added more significant insights into the pathological and diagnostic aspects of MCADD, which should be integrated with the general knowledge driven by NBS and genetic testing to provide a complementary understanding of MCADD and its pathology. Our study gives a metabolomics basis for future studies that are focused on MCADD. It also helps clinical scientists and physicians visualize a larger picture of the pathophysiology of MCADD, which could assist in the improved diagnosis and treatment of MCADD.

## Figures and Tables

**Table 1:** Demographic data of MCADD newborns and healthy controls.

Group		MCADD	Healthy Control	*p*-Value
Number		14	14	NA
Age average (Day)		15.3	11	0.2710
Female (%)		78%	71%	NA
Data	C8\C10-carnitine ratio (cutoff: <1.6)	15.2	<1.6	5.30 × 10^−6^
	C8-carnitine (cutoff: <0.32 µM)	2.1	<0.32	0.0007
	C6-carnitine (cutoff: <0.35 µM)	0.6	<0.35	0.0004
	C8\C2-carnitine ratio (cutoff: <0.1)	0.3	<0.1	0.04

For statistical analyses, an unpaired student *t*-test was conducted. Significance is considered when the *p*-value < 0.05.

## Data Availability

The raw data of this study were deposited to Metabolomics Work- bench and can be accessed at (accession number ST002557) on 28 April 2023.

## Supplementary Materials

The supporting information can be downloaded at: https://www.mdpi.com/article/10.3390/ijms24119657/s1.
