# Supplementary material for: Untargeted Metabolomics Identifies Biomarkers for MCADD Neonates in Dried Blood Spots
Source: Int J Mol Sci. 2023 Jun 2;24(11):9657. doi: 10.3390/ijms24119657 (PMC10253986; doi:10.3390/ijms24119657)
Supplement: Supplementary file 1 [file ijms-24-09657-s001.zip › ijms-2362896-supplementary/Supplementary figure 1.pdf]

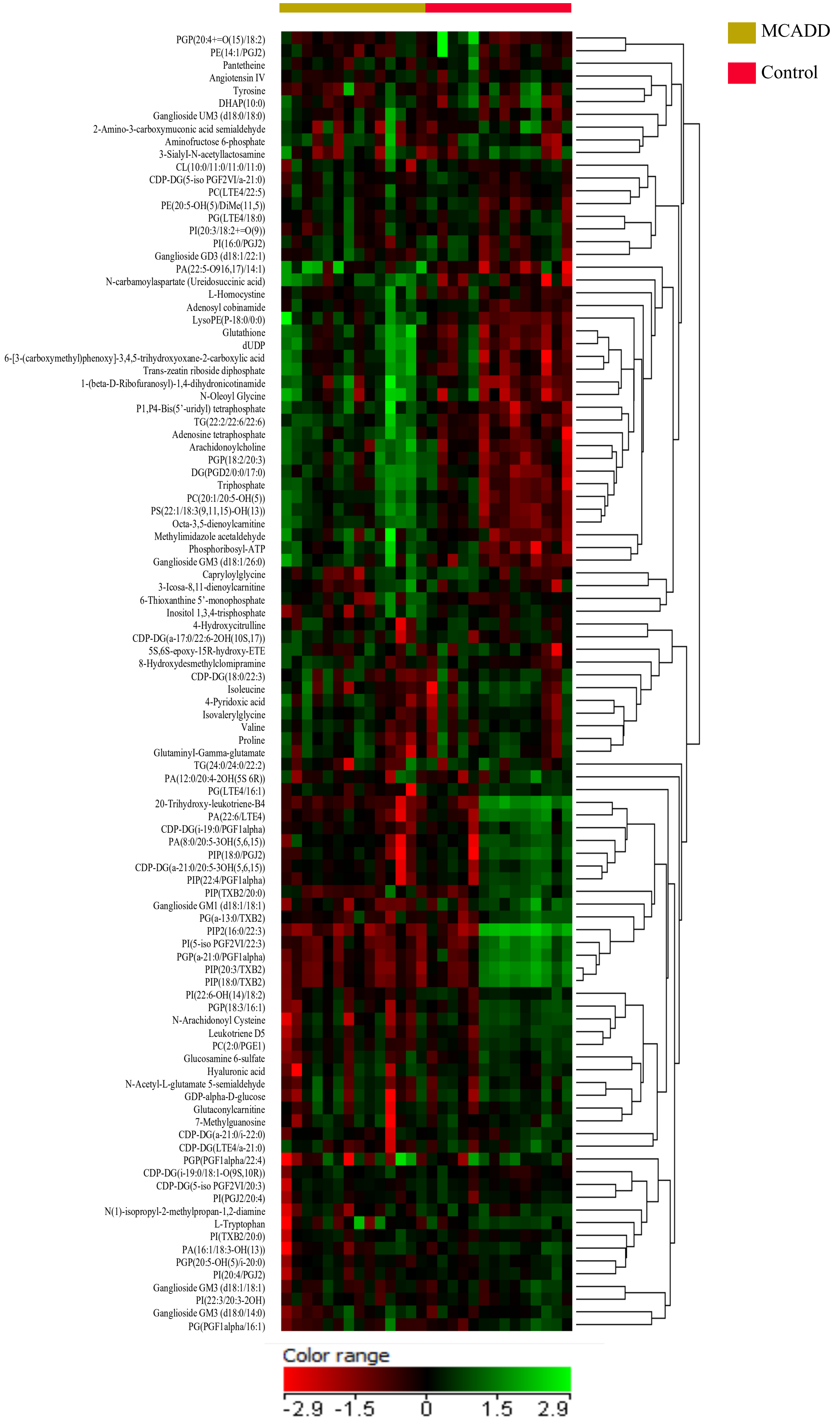

MCADD
Control
PGP(20:4+=O(15)/18:2)
PE(14:1/PGJ2)
Pantetheine
Angiotensin IV
Tyrosine
DHAP(10:0)
Ganglioside UM3 (d18:0/18:0)
2-Amino-3-carboxymuconic acid semialdehyde
Aminofructose 6-phosphate
3-SialyI-N-acetyllactosamine
CL(10:0/11:0/11:0/11:0)
CDP-DG(5-iso PGF2VI/a-21:0)
PC(LTE4/22:5)
PE(20:5-OH(5)/DiMe(11,5))
PG(LTE4/18:0)
PI(20:3/18:2+=O(9))
PI(16:0/PGJ2)
Ganglioside GD3 (d18:1/22:1)
PA(22:5-O916,17)/14:1)
N-carbamoylaspartate (Ureidosuccinic acid)
L-Homocystine
Adenosyl cobinamide
LysoPE(P-18:0/0:0)
Glutathione
dUDP
6-[3-(carboxymethyl)phenoxy]-3,4,5-trihydroxyoxane-2-carboxylic acid
Trans-zeatin riboside diphosphate
1-(beta-D-Ribofuranosyl)-1,4-dihydronicotinamide
N-Oleoyl Glycine
P1,P4-Bis(5’-uridyl) tetraphosphate
TG(22:2/22:6/22:6)
Adenosine tetraphosphate
Arachidonoylcholine
PGP(18:2/20:3)
DG(PGD2/0:0/17:0)
Triphosphate
PC(20:1/20:5-OH(5))
PS(22:1/18:3(9,11,15)-OH(13))
Octa-3,5-dienoylcarnitine
Methylimidazole acetaldehyde
Phosphoribosyl-ATP
Ganglioside GM3 (d18:1/26:0)
Capryloylglycine
3-Icosa-8,11-dienoylcarnitine
6-Thioxanthine 5’-monophosphate
Inositol 1,3,4-trisphosphate
4-Hydroxycitrulline
CDP-DG(a-17:0/22:6-2OH(10S,17))
5S,6S-epoxy-15R-hydroxy-ETE
8-Hydroxydesmethylclomipramine
CDP-DG(18:0/22:3)
Isoleucine
4-Pyridoxic acid
Isovalerylglycine
Valine
Proline
Glutaminyl-Gamma-glutamate
TG(24:0/24:0/22:2)
PA(12:0/20:4-2OH(5S 6R))
PG(LTE4/16:1)
20-Trihydroxy-leukotriene-B4
PA(22:6/LTE4)
CDP-DG(i-19:0/PGF1alpha)
PA(8:0/20:5-3OH(5,6,15))
PIP(18:0/PGJ2)
CDP-DG(a-21:0/20:5-3OH(5,6,15))
PIP(22:4/PGF1alpha)
PIP(TXB2/20:0)
Ganglioside GM1 (d18:1/18:1)
PG(a-13:0/TXB2)
PIP2(16:0/22:3)
PI(5-iso PGF2VI/22:3)
PGP(a-21:0/PGF1alpha)
PIP(20:3/TXB2)
PIP(18:0/TXB2)
PI(22:6-OH(14)/18:2)
PGP(18:3/16:1)
N-Arachidonoyl Cysteine
Leukotriene D5
PC(2:0/PGE1)
Glucosamine 6-sulfate
Hyaluronic acid
N-Acetyl-L-glutamate 5-semialdehyde
GDP-alpha-D-glucose
Glutaconylcarnitine
7-Methylguanosine
CDP-DG(a-21:0/i-22:0)
CDP-DG(LTE4/a-21:0)
PGP(PGF1alpha/22:4)
CDP-DG(i-19:0/18:1-O(9S,10R))
CDP-DG(5-iso PGF2VI/20:3)
PI(PGJ2/20:4)
N(1)-isopropyl-2-methylpropan-1,2-diamine
L-Tryptophan
PI(TXB2/20:0)
PA(16:1/18:3-OH(13))
PGP(20:5-OH(5)/i-20:0)
PI(20:4/PGJ2)
Ganglioside GM3 (d18:1/18:1)
PI(22:3/20:3-2OH)
Ganglioside GM3 (d18:0/14:0)
PG(PGF1alpha/16:1)
Color range
-2.9 -1.5 0 1.5 2.9

**Figure S1:** Heatmap showing the expression levels of 107 dysregulated metabolites in MCADD newborns compared with healthy controls. Red indicates downregulated metabolites, and green indicates upregulated metabolites.
